# Supplementary material for: Rethinking glottal midline detection
Source: Sci Rep. 2020 Nov 26;10:20723. doi: 10.1038/s41598-020-77216-6 (PMC7693305; doi:10.1038/s41598-020-77216-6)
Supplement: Supplementary file 1 — Supplementary Information. [file 41598_2020_77216_MOESM1_ESM.pdf]

Supplementary Information (SI)

Rethinking glottal midline detection

Andreas M. Kist[1,*], Julian Zilker[1], Pablo Gómez[1], Anne Schützenberger[1], Michael Döllinger[1]

[1]Division of Phoniatrics and Pediatric Audiology, Department of Otorhinolaryngology, Head and Neck Surgery, University Hospital Erlangen, Friedrich-Alexander-University Erlangen-Nürnberg, 91054 Erlangen, Germany

* corresponding author: andreas.kist@uk-erlangen.de

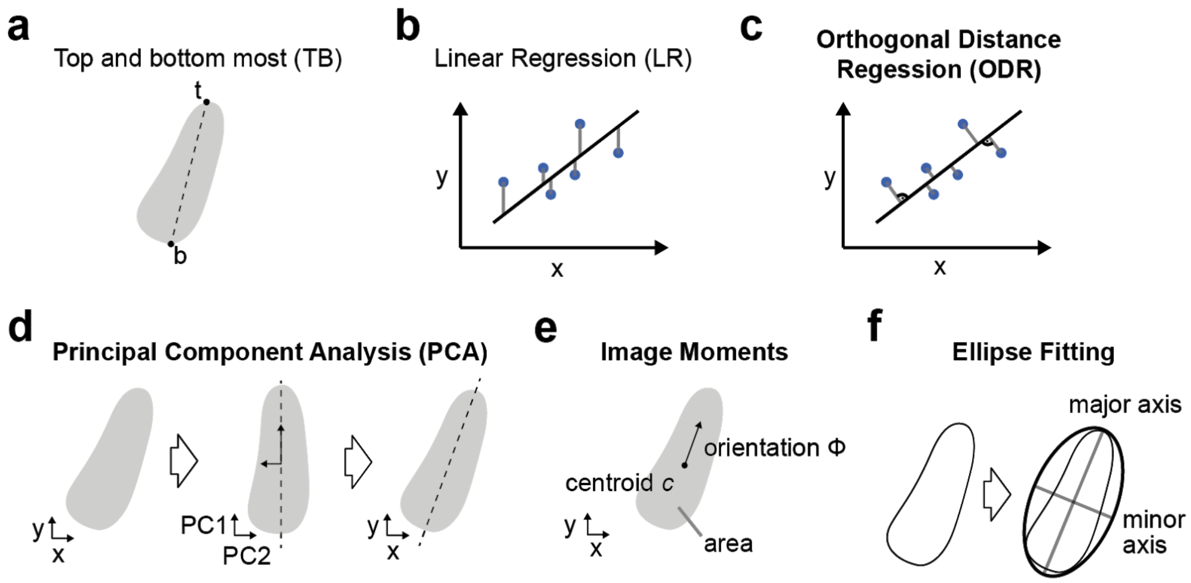

Supplementary Figure 1. Overview of computer vision algorithms. Algorithms described first in this study have bold headings. **a)** Top and bottom most point, **b)** linear regression, **c)** orthogonal distance regression, **d)** principal component analysis, **e)** image moments, **f)** ellipse fitting.

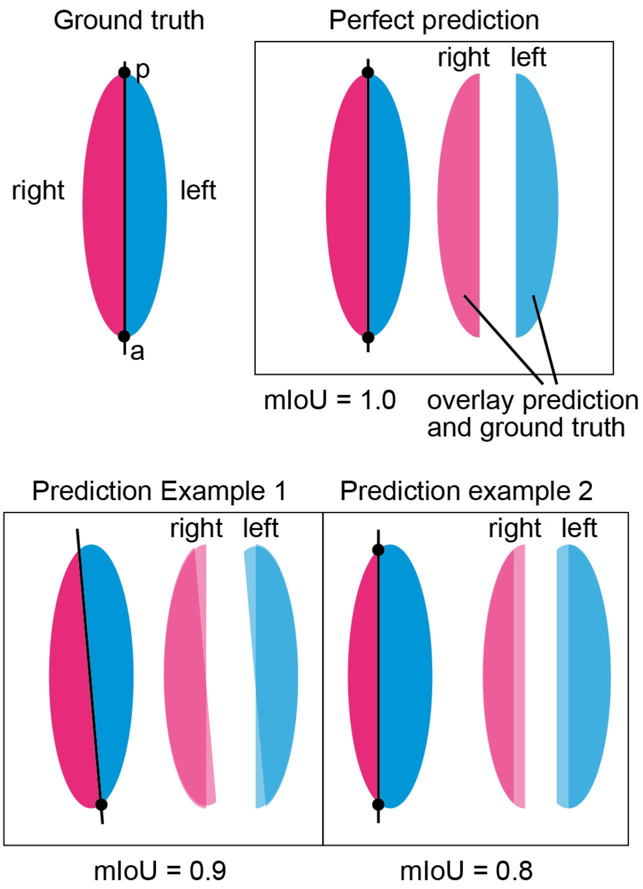

Supplementary Figure 2. Computation of the mean Intersection over Union (mIoU). The area is divided by the midline (connection of points p and a) into left and right. The same total area is divided by the predicted p and a points resulting in potentially different left and right areas. The mean intersection over the union of the two areas is computed to evaluate the goodness of the prediction.

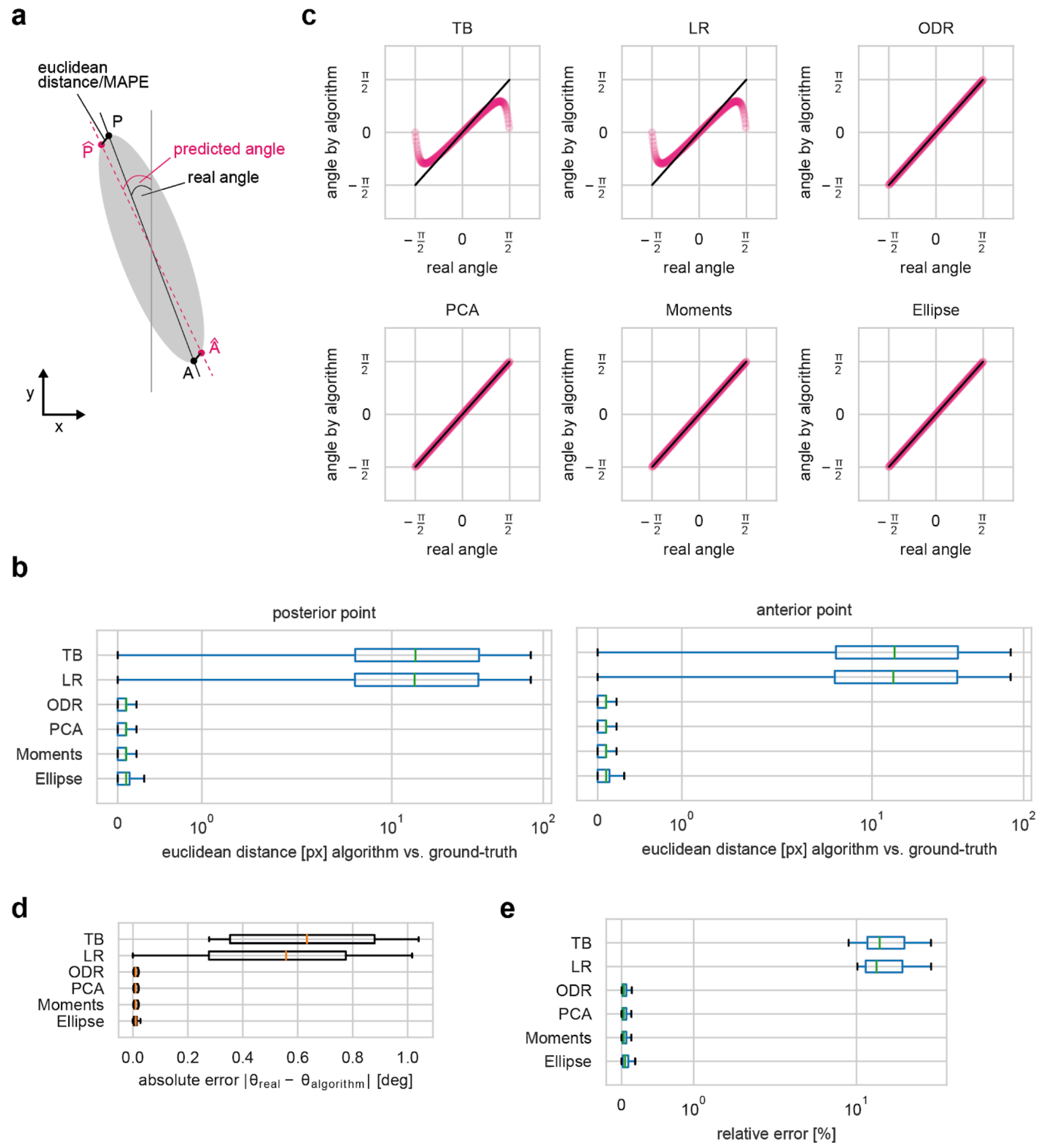

Supplementary Figure 3. Computer vision performance on a toy dataset. a) Toy dataset setup. A binary ellipse is moved out of center and rotated from -90° (-0.5 π) to +90° (+0.5 π). The difference between the real and the predicted angle as well as the distances between predicted and real posterior and anterior point are computed (^P vs. P and Â vs. A, respectively). **b)** Absolute differences between algorithm and ground-truth posterior and anterior point. **c)** Relationship between predicted angle and real angle across algorithms. **d)** Absolute deviation of predicted and real angle across algorithms. **e)** Relative error of predicted angles (as shown in d).

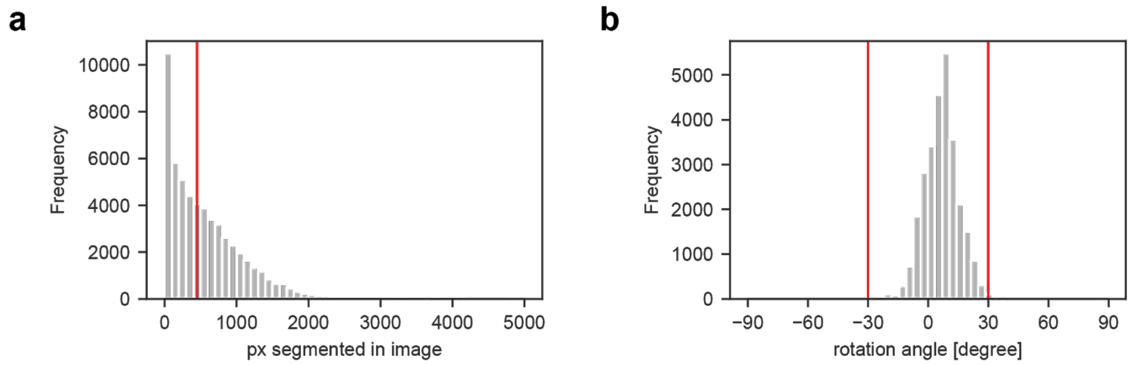

Supplementary Figure 4: Glottis rotation angles in the BAGLS dataset. a) Distribution of segmented px across the BAGLS dataset. The median of segmented px is indicated by a red vertical line (median = 452 segmented px). **b)** Distribution of rotation angles determined by PCA of segmentation maps that contain at least 452 px. Red vertical lines indicate the rotation angle borders used for 6MM augmentation.

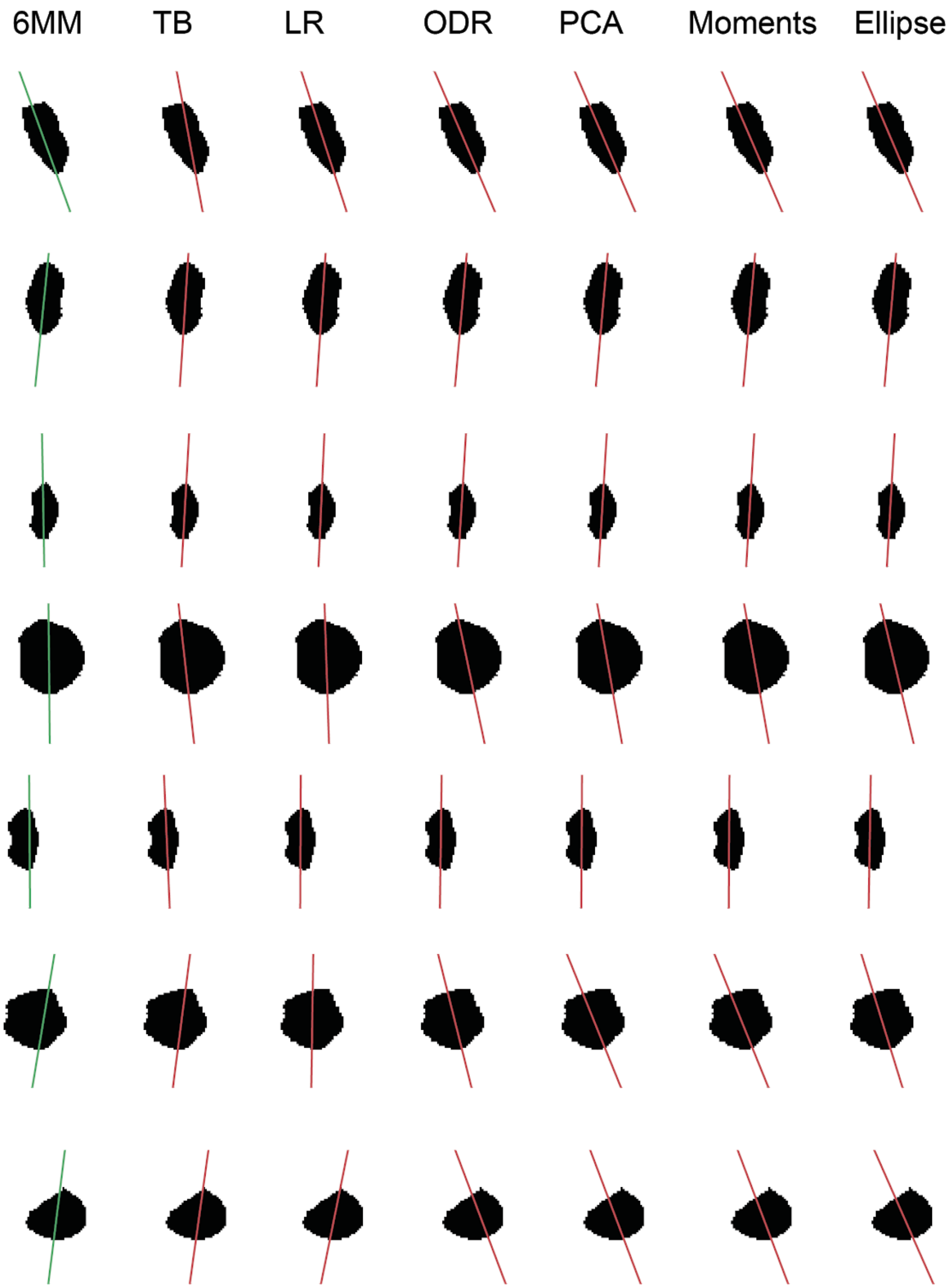

Supplementary Figure 5. Comparison 6MM with computer vision algorithms. Examples of different 6MM simulations (shown is maximum opening of a given cycle) together with the ground-truth midline (green) and the respective predictions using the evaluated algorithms, i.e. TB; LR, ODR, PCA, image moments and ellipse fitting.

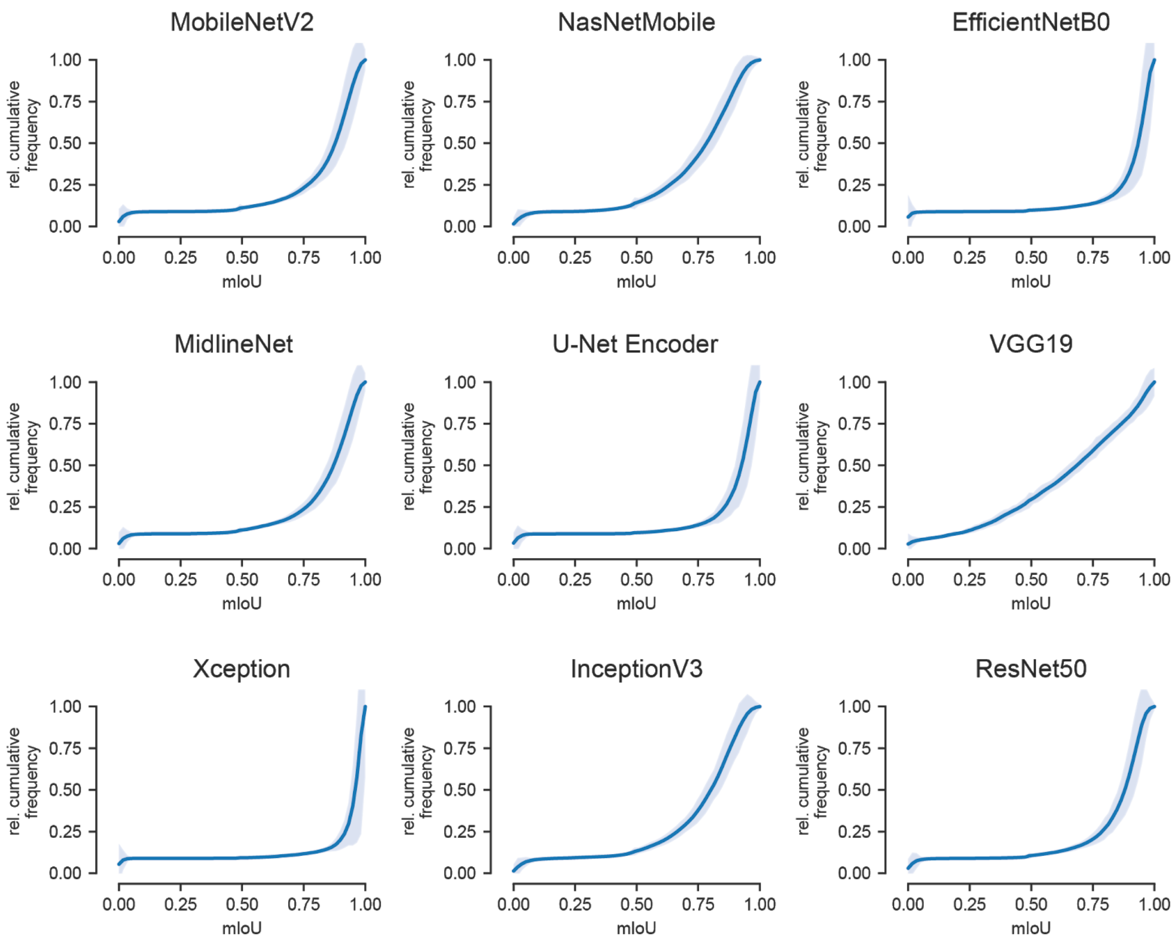

Supplementary Figure 6: Performance of individual neural networks on predicting the glottal midline in 6MM data

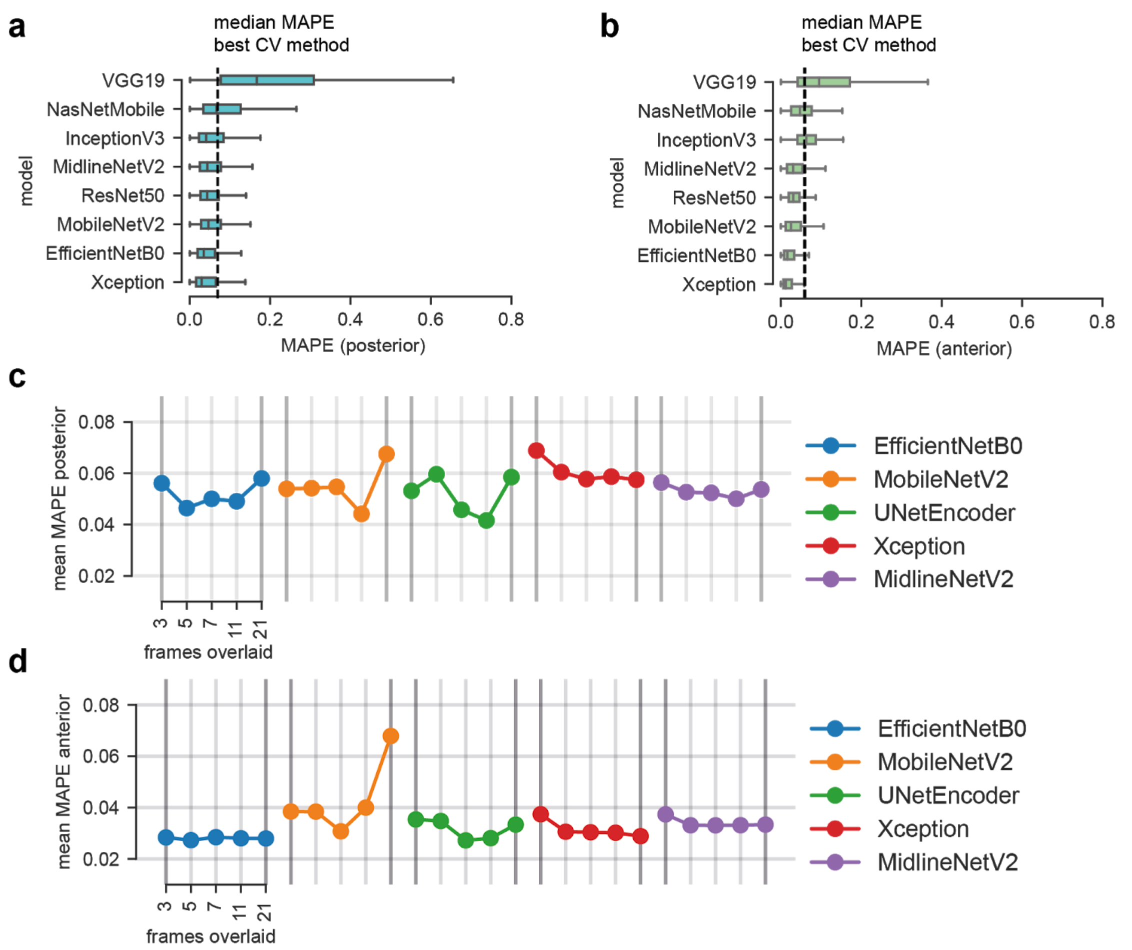

Supplementary Figure 7: MAPE distribution of neural networks. a) MAPE for posterior point, **b)** MAPE for anterior point**, c)** MAPE for posterior point with temporal context**, d)** MAPE for anterior point with temporal context.

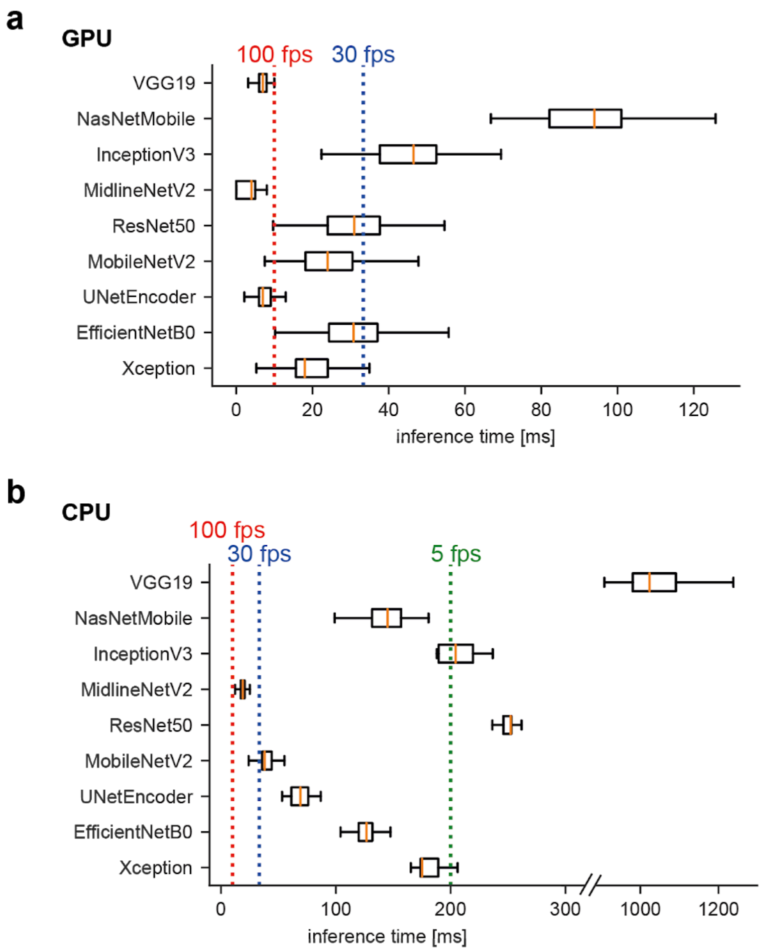

Supplementary Figure 8: Neural network inference time. Inference time was measured in a) on a Titan RTX GPU, and in b) on an Intel Xeon Silver 4116 CPU @ 2.10 GHz.

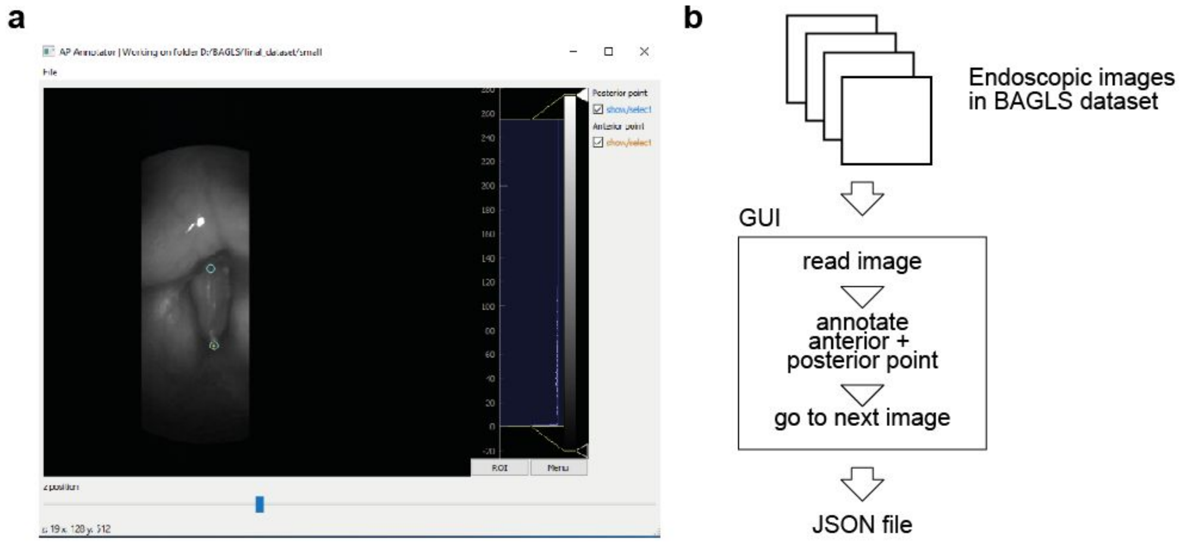

Supplementary Figure 9. Annotating tool. a) Screenshot from the new annotating tool. **b)** Schematic workflow. The software relies on Python together with PyQt5 and pyqtgraph for fast image plotting and ROI annotation. ROIs are stored in JSON format. The tool is freely available upon publication.

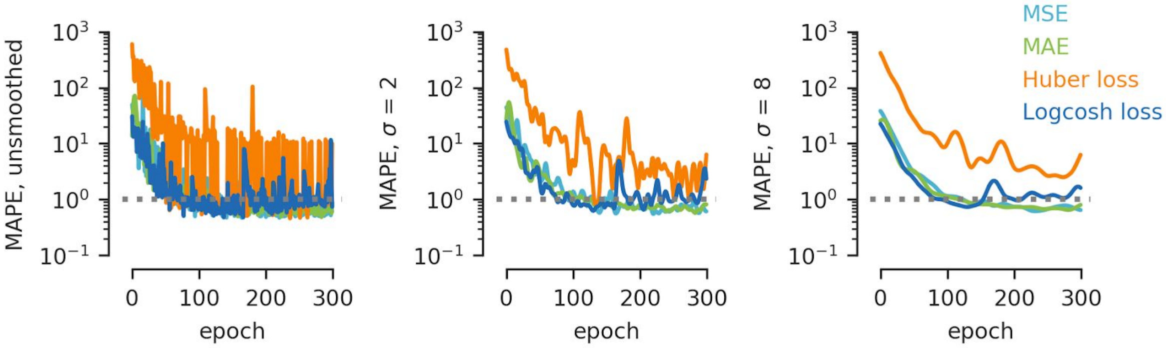

Supplementary Figure 10. Performance of different losses. We tested mean absolute error (MAE), mean squared error (MSE), Logcosh loss and Huber loss. Data was either unsmoothed or smoothed with a Gaussian filter, where σ indicates the kernel width.

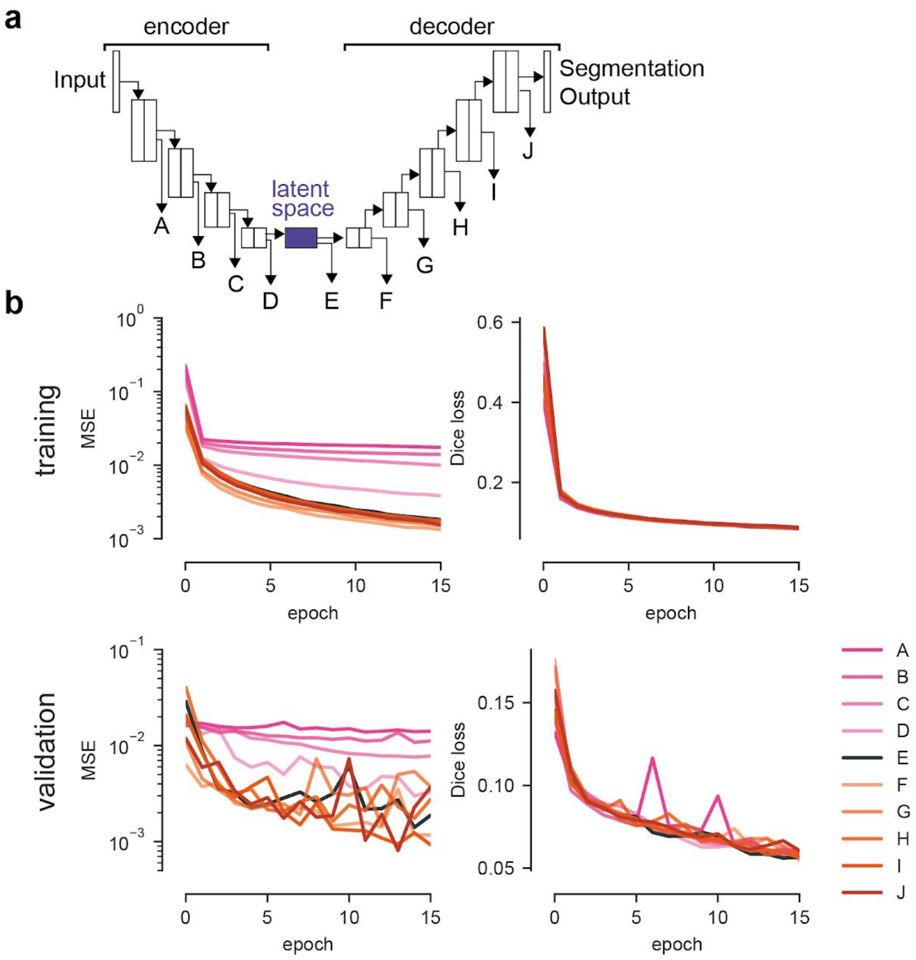

Supplementary Figure 11. Evaluation of anterior and posterior point prediction site.

a) Encoder-Decoder scheme with endoscopic image (input) and the segmentation map as output. All evaluated prediction sites are labelled A-J. **b)** Training and validation performance of evaluated prediction sites (left panels, mean squared error, MSE) and segmentation (right panels, dice loss). Color coded in encoder (shades of pink), latent space (black) and decoder (shades of orange). Note the coherent performance in segmentation (as expected) and the differences in anterior and posterior point prediction.

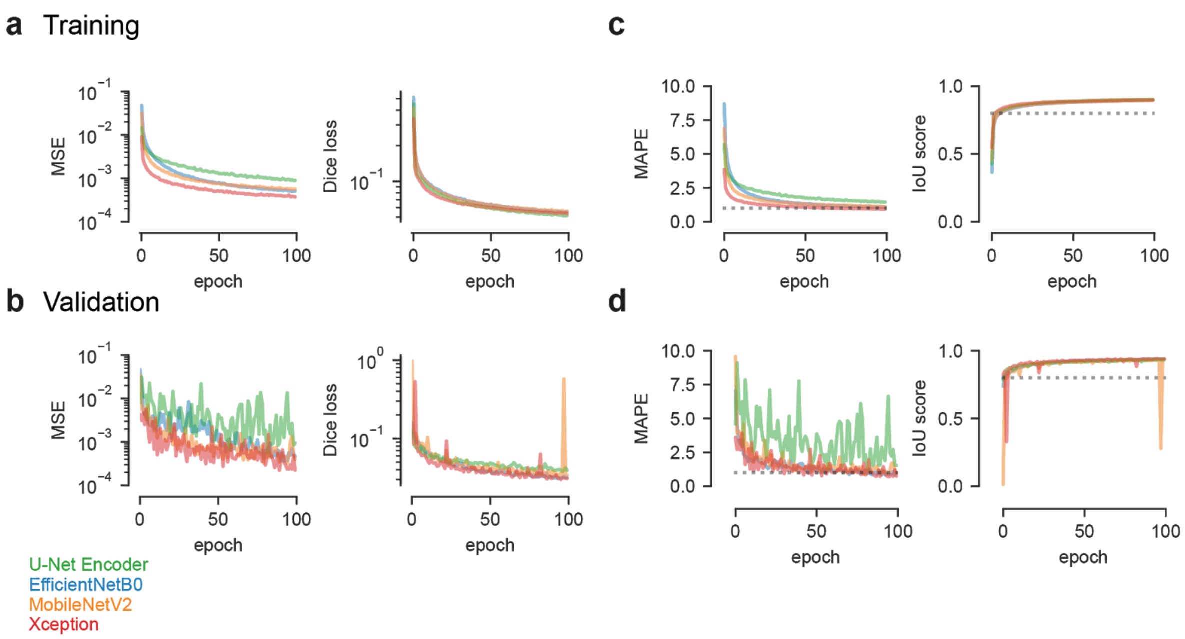

Supplementary Figure 12. GlottisNet performance with different backbones. a) Training loss for different encoder backbones (EfficientNetB0 in blue, MobileNetV2 in orange and U-Net in green, color code applies to all other panels) for midline detection (MSE loss, left panel) and segmentation (Dice loss, right panel). **b)** Validation loss, color code and loss as in a). **c)** Performance measures for midline detection (mean absolute percentage error, MAPE, left panel) and segmentation (intersection over union (IoU) score, right panel), same color code as in a) and b). **d)** Performance measures as in c) for validation set.

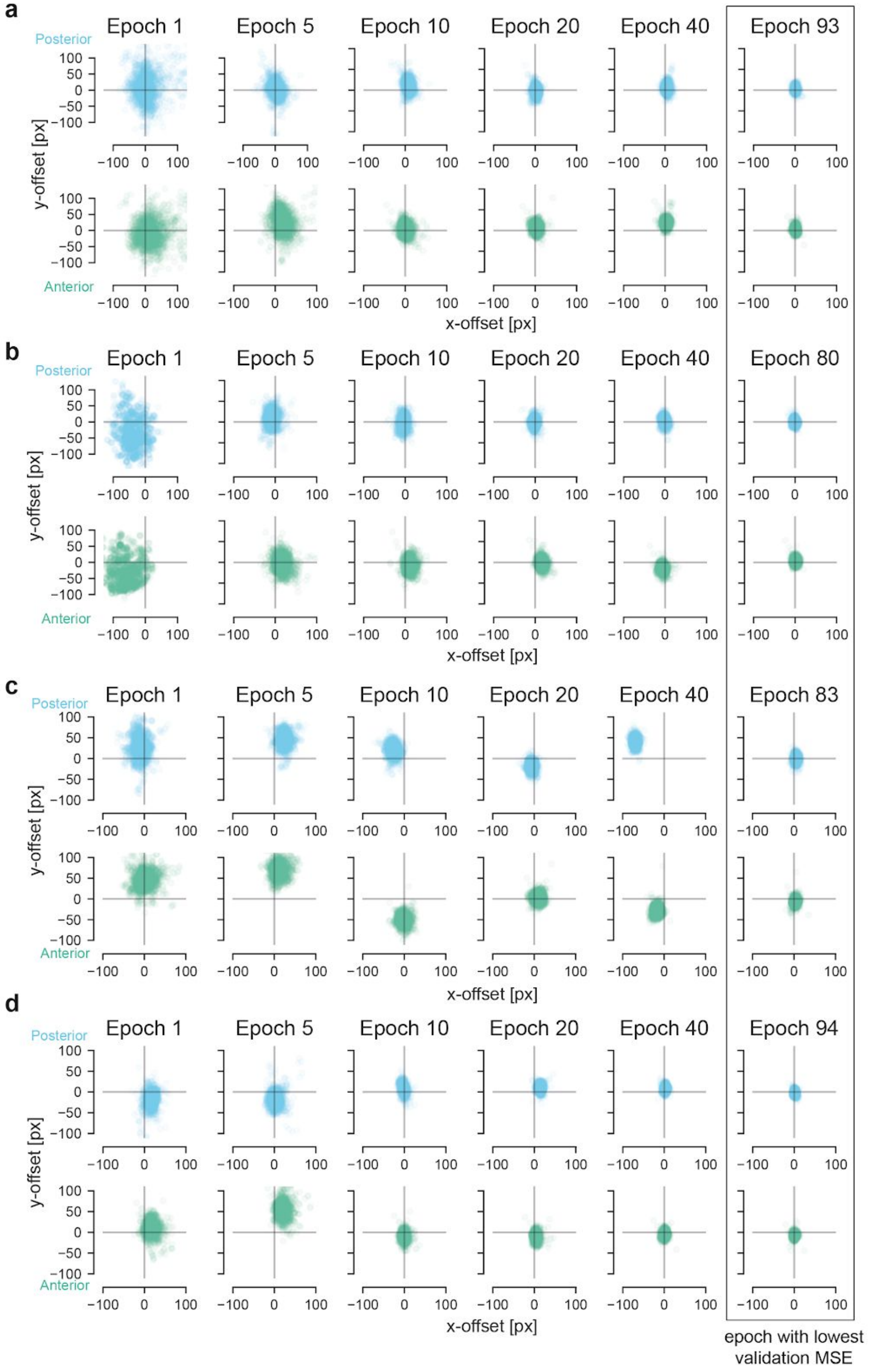

Supplementary Figure 13. Convergence of neural networks over time. a) EfficientNetB0 encoder, **b)** MobileNetV2 encoder, **c)** U-Net encoder, **d)** Xception encoder

Supplementary Videos

Supplementary Movie 1.
High-speed videoendoscopy footage from healthy, oscillating vocal folds.

Supplementary Movie 2.
Result of numerical simulation using the 6MM model together with its respective glottal area waveform. A symmetric and an asymmetric example is shown.

Supplementary Movie 3.
Performance of investigated computer vision methods on predicting the midline of a binary, rotating ellipse.

Supplementary Movie 4.
Performance of GlottisNet on various high-speed endoscopy video footage.

Supplementary Methods

1 1D Six-Mass-Model

1.1 Variables

(s, i) - $i = 1$ to $i = 10$, lower/upper ($s = 1, s = 2$)
$m_{s,i}$ - masses $i = 1$ to $i = 5$ on the left, 6 to 10 on the right respectively from top to bottom
$k^l_{s,i}$ - stiffness of the longitudinal coupling springs between mass s, i and $s, i+1$
$k^a_{s,i}$ - stiffness of the anchor spring at mass s, i
k^v_i - stiffness of the vertical coupling spring between masses $1, i$ masses $2, i$
$k^c_{s,i}$ - restoring spring stiffness during collision
$r^a_{s,i}$ - anchor force damping coefficients
$x_{s,i}$ - position of the mass in relation to the rest position
$x^r_{s,i}$ - rest position of the mass $m_{s,i}$
$x^p_{s,i} = x^r_{s,i} + x_{s,i}$ - position of the mass in the global coordinate system
$L_{s,i} = (x^p_{s,i+5} - x^p_{s,i})$ - distance between mass s, i on the left and $s, i+5$ on the right
P_{sub} - subglottal pressure
d - distance between the planes $s = 1$ and $s = 2$
l - glottis length
$\Lambda_d(x) \approx \sqrt{x^2 + d^2} - d$ - approximates vertical distance of masses using Lagrange interpolation
$\Lambda_l(x) \approx \sqrt{x^2 + (\frac{l}{4})^2} - \frac{l}{4}$ - approximates vertical distance of masses using Lagrange interpolation

1.2 Forces

$$m_{s,i}\ddot{x}_{s,i} = F^a_{s,i} + F^v_{s,i} + F^l_{s,i} + F^c_{s,i} + F^d_{s,i} \quad (1)$$

where

$F^a_{s,i}$ - anchor spring force
$F^v_{s,i}$ - vertical coupling force
$F^l_{s,i}$ - longitudinal coupling force
$F^c_{s,i}$ - force due to collision
$F^d_{s,i}$ - driving force

s. t.

$$\ddot{x}_{s,i} = \left(\frac{F^a_{s,i} + F^v_{s,i} + F^l_{s,i} + F^c_{s,i} + F^d_{s,i}}{m_{s,i}} \right) \tag{2}$$

1.2.1 Anchor Spring Force

$$F^a_{s,i} = -k^a_{s,i} x_{s,i} - r^a_{s,i} \dot{x}_{s,i} \tag{3}$$

1.2.2 Vertical Coupling Force

$$F^v_{s,i} = -k^v_i \Lambda_d (x_{s,i} - x_{s^*,i}) \tag{4}$$

with

$$s^* = 3 - s \quad \text{, i.e. the lower/upper corresponding index}$$

1.2.3 Longitudinal Coupling Force

$$\begin{aligned} F^l_{s,i} = & -k^l_{s,i-1} \Lambda_l (x_{s,i} - x_{s,i-1}) \\ & -k^l_{s,i} \quad \Lambda_l (x_{s,i} - x_{s,i+1}) \end{aligned} \tag{5}$$

with

$$k^l_{s,j} = \xi_k (k^a_{s,j} + k^a_{s,j+1}), \quad \xi_k = 0.2 \tag{6}$$

1.2.4 Driving Force

$$F^d_{1,i} = \frac{(-1)^\delta P_i l d}{3} \tag{7}$$

$$\delta = \begin{cases} 1 & \text{when} \quad i \leq 5 \\ 0 & \text{else} \end{cases}$$

$$P_i = \begin{cases} P_{sub}\left(1 - \Theta(a_{min,i)}\frac{L^2_{min,i}}{L^2_{1,i}}\right)\Theta(L_{1,i}) & \text{for} \quad 2 \leq i \leq 4 \\ P_{i-5} & \text{for} \quad 7 \leq i \leq 9 \\ 0 & \text{else} \end{cases} \tag{8}$$

$$a_{min,i} = \min(a_{1,i}, a_{2,i})$$

1.2.5 Collision Force

$$F^c_{s,i} = \begin{cases} -\Theta(-L_{s,i})(k^c_{s,i} + k^c_{s,i+5})\frac{L_{s,i}}{16} & \text{when} \quad i \leq 5 \\ -F^c_{s,i-5} & \text{else} \end{cases} \tag{9}$$
